# Association of serum uric acid with functional disability in older subjects: a population-based study

**DOI:** 10.1007/s40520-024-02746-2

**Published:** 2024-04-17

**Authors:** Alice Laudisio, Agnese Dorizzi, Fabio Villeggia, Francesca Latino, Daniele Filippucci, Giuseppe Zuccalà

**Affiliations:** 1 Department of Medicine, Research Unit of Geriatrics, Università Campus Bio-Medico di Roma, Via Alvaro del Portillo, Roma, 21 - 00128 Italy; 2 Operative Research Unit of Geriatrics, Fondazione Policlinico Universitario Campus Bio-Medico, Via Alvaro del Portillo, Roma, 200 - 00128 Italy; 3 Department of Geriatric and Orthopaedic Sciences, Catholic University of Medicine, Rome, Italy; 4 Fondazione Policlinico Universitario A. Gemelli IRCCS, Largo F. Vito 1, Rome, 00168 Italy

**Keywords:** Serum uric acid, Disability, Elderly

## Abstract

**Background:**

The role of serum uric acid (SUA) in the development of adverse health outcomes in advanced age is still uncertain.

**Aims:**

The aim of the study was to assess the association of disability with SUA levels in older community-dwelling subjects.

**Methods:**

We assessed the association of disability with SUA in all 351 inhabitants of Tuscania (Italy) aged 75+. Functional ability was estimated using the instrumental activities of daily living (IADLs).

**Results:**

In logistic regression, increasing SUA levels were associated with disability (OR = 1.22; 95%CI = 1.01–1.48; *P* = .036), after adjusting. The association was independent of both gender and age (P for interaction > 0.050). SUA levels above 5.5 mg/dL best predicted disability.

**Conclusions:**

In older subjects, SUA levels are associated with disability; the cut off level above 5.5 mg/dL might be adopted in pharmacological trials aiming at reducing the incidence and progression of disability by reducing SUA, and for identifying subjects at increased risk of disability.

## Introduction

The prevalence rates of hyperuricemia are increasing worldwide, possibly reflecting the progressive aging of western populations, as well as changes in lifestyle habits [1]. Serum uric acid (SUA) is currently considered a biologically active compound with complex, often contrasting effects on health and disease. In fact, some experimental studies have suggested that SUA might modulate aging processes, enhancing the resistance to oxidative stress and possibly extending the life span of invertebrate, as well mammalian models.^

However, an increasing bulk of epidemiological and experimental data indicate that SUA is closely related to cardiovascular risk and events. Moreover, SUA-lowering therapy by xanthine oxidase inhibitors has led to improved cardiovascular outcomes in clinical, as well as experimental studies.

Disability represents an increasing threat for the sustainability of healthcare and social systems in western aging countries. In 2022, 27% of the EU population over the age of 16 had some form of disability; according to Eurostat estimates, that equals to 101 million people, or one in four people adults in the EU. As expected, European data indicate that prevalent disability increases with advancing age, from 8% in the age strata 16–19 years to over 52% among people aged 65+. Disability is associated with decreased survival and quality of life; also, it represents a major source of healthcare and social expenditures: according to Eurostat data, In 2018, the EU Member States spent approximately €276 billion on disability benefits. Not surprisingly, several large multicenter trials have been devoted to the prevention, or at least delay, of disability [2]. In addition, the search for new risk factors and pathways for the loss of functional ability has been promoted [2].

Elevated SUA levels have been associated with increased risk of cerebrovascular events, including stroke and myocardial infarction. Also, increased SUA levels are associated with increased incidence of peripheral polyneuropathy among patients with type 2 diabetes, and represent a risk factor for incident osteoarthritis. Noticeably, all these conditions are associated with disability [3]. Thus, increased SUA might represent a potentially amendable cause of disability in older populations.

Disability has been adopted as primary outcome by several large epidemiological studies and clinical trials. This is justified by the role of disability as a major cause of reduced survival and quality of life, as well as increased healthcare and social costs. This study aims at assessing the association between SUA levels and disability in a whole population of community-dwelling elderly.

## Methods

### Participants

The study involved all 387 residents aged 75 or older who were living in the town of Tuscania (Italy) on January 1st, 2014. These participants had been enrolled in a national study on the genetic determinants of health status in six towns [4]. Among those 387 participants, we excluded 11 subjects on allopurinol or febuxostat, and 25 subjects with missing data; thus, data for the present study were available for 351 subjects. Excluded participants did not differ significantly from those included.

The Institutional Review Board of the Catholic University approved the protocol of the study, and all patients provided an informed consent.

Functional ability was self-reported, measured by the Lawton and Brody scale for instrumental activities of daily living (IADLs) [5]. Impairment in IADL function was defined by a score < 7; this higher cutoff level for identifying functional disability is generally adopted to avoid a “floor effect”.

Blood samples were obtained after overnight fast; SUA levels were measured using the uricase HMMPS method on a Roche analyzer (Cobas 8000-c702; Roche Diagnostics, Switzerland), and expressed as mg/dL.

Smoking was calculated as total lifetime pack years for current and former smokers. Current alcohol consumption was considered for at least two drinks per week.

The burden of comorbidity was quantified using the Charlson comorbidity index score.

Cognitive performance was assessed using the Hodkinson Abbreviated Mental Test (AMT). Dietary habits were evaluated using the Mediterranean Diet Score; social vulnerability was explored using the Gijon’s social-familial evaluation scale.

Statistical analyses were performed using IBM SPSS (26.0) statistical software. Differences were considered significant at the *P* < .050 level. Data of continuous variables are presented as mean values ± standard deviation (SD). Medians and inter-quartile ranges were provided for non-normally distributed variables. Analysis of variance (ANOVA) for normally distributed variables was performed according to the presence of disability; otherwise, the nonparametric Mann-Whitney U test was adopted. Chi square with the two-tailed Fisher exact test was used for dichotomous variables.

Multivariable logistic regression analysis was used to assess the association of disability with age, sex, SUA levels, and all those variables which differed significantly (*P* < .050) in univariate analyses. Abnormally distributed variables were analyzed after log transformation.

Analysis of the interaction terms “SUA*age” and “SUA*gender” was performed to assess whether the association of functional disability with uric acid varied according to these demographic characteristics.

Eventually, ROC curve analysis was adopted to estimate the predictive value of SUA levels, through the evaluation of the Areas Under the Curve; the optimal uric acid cutoff value was determined as the point on the ROC curve that maximized the Youden Index.

## Results

Disability was recorded in 128 (36%) participants. The mean SUA level was 5.5 (± 1.6) mg/dL. Of notice, disabled subjects had higher uric acid levels (i.e., 5.8 (2) vs. 5.3 (1.4) mg/dL, *P* = .003).

The characteristics of participants according to the presence of disability are shown in Table 1.

**Table 1:** Characteristics of 351 participants according to the presence of disability (IADLs score < 7)

	Disabled subjects (*n* = 128)			Controls (*n* = 223)	P
	***prevalence, %, or mean (SD) or median (IQR range)***				
***Demographics***					
Age (years)	82 (5)			78 (5)	< 0.0001
Gender (female)	61 (48)			131 (59)	0.046
Education (years)	5 (3–5)			5 (3–8)	0.103
Smoking (total lifetime pack-years)	9490 (5475–12,775)			7774 (3832–16,060)	0.540
Current alcohol consumption	89 (69)			158 (71)	0.809
Mediterranean Diet Score	4 (1)			5 (1)	0.924
Social-familial evaluation scale	10 (3)			9 (2)	0.626
***Comorbid conditions***					
Diabetes	39 (30)			40 (18)	0.008
Hypertension	92 (72)			146 (65)	0.237
Heart failure	40 (31)			25 (11)	< 0.0001
Coronary disease	30 (23)			36 (16)	0.118
Stroke or Transient Ischemic Attacks	30 (23)			15 (7)	< 0.0001
Renal disease	9 (7)			7 (3)	0.112
Malignancy	14 (11)			19 (8)	0.454
Hepatic disease	6 (5)			8 (4)	0.586
Peripheral artery disease	9 (7)			8 (4)	0.196
Parkinson’s disease	9 (7)			4 (2)	0.018
Arthritis	97 (76)			178 (80)	0.420
Charlson score	3 (2–5)			1 (0–2)	< 0.0001
***Medications***					
Beta-blockers	6 (5)			11 (5)	0.999
Corticosteroids	5 (4)			9 (4)	0.999
Statins	5 (4)			29 (13)	0.005
Antiplatelets	33 (26)			39 (17)	0.074
Anticoagulants	8 (6)			11 (5)	0.629
SSRI	7 (5)			6 (3)	0.241
Sartans	33 (26)			39 (17)	0.074
ACE-inhibitors	39 (30)			66 (29)	0.904
NSAIDs	10 (8)			15 (7)	0.830
Loop diuretics	39 (30)			29 (13)	< 0.0001
Benzodiazepines	33 (26)			39 (17)	0.074
***Biohumoral parameters***					
Creatinine (mg/dL)	1.1 (0.5)			0.9 (0.2)	< 0.0001
Albumin (g/dL)	4.0 (0.6)			4.3 (0.6)	< 0.0001
Hemoglobin (g/dl)	13.8 (1.9)			14.4 (1.5)	0.005
Total cholesterol (mg/dL)	199 (42)			220 (40)	< 0.0001
Uric acid (mg/dL)	5.8 (2.0)			5.3 (1.4)	0.003
***Physical and cognitive parameters***					
Body Mass Index	28.8 (5.1)			28.1 (4.6)	0.212
Hodkinson Abbreviated Mental Test	7 (3)			9 (1)	< 0.0001

In logistic regression, disability was associated with higher SUA levels in the unadjusted model (OR = 1.22; 95% CI = 0.1.07–1.40; *P* = .003), after adjusting for age and sex (OR = 1.20; 95% CI = 0.1.04–1.38; *P* = .015), and in the final model (OR = 1.22; 95% CI = 1.01–1.48; *P* = .036, Table 2), adjusted for age, sex, and those variables significantly associated in univariate models (i.e. diabetes, heart failure, stroke, and Parkinson’s disease, Charlson, and AMT score, use of statins, and loop diuretics, hemoglobin, albumin, cholesterol levels).

**Table 2:** Association (Odds Ratios. OR, and 95% confidence intervals, CI) between functional disability (i.e. IADLs score < 7) and the variables of interest according to logistic regression analysis. All the covariates were entered simultaneously into the regression model

	OR	95% CI	P
Age (each year)	1.05	0.99–1.123	0.103
Sex (female)	0.36	0.18 − 0.73	0.005
Diabetes	0.89	0.78–1.01	0.068
Heart failure	0.54	0.23–1.28	0.163
Stroke or Transient Ischemic Attacks	1.32	1.12–1.58	0.022
Parkinson’s disease	0.45	0.11–1.73	0.244
Charlson comorbidity score index	1.18	0.94–1.47	0.154
Statins	0.30	0.09 − 0.99	0.050
Serum albumin (g/dL)	0.49	0.28 − 0.87	0.014
Hemoglobin (g/dL)	0.90	0.73–1.12	0.337
Total cholesterol (mg/dL)	0.99	0.99–1.02	0.152
Hodkinson Abbreviated Mental Test	0.62	0.51 − 0.76	< 0.0001
Serum acid uric (mg/dL)	1.22	1.01–1.48	0.036

Analysis of the interaction term indicated that the association of disability with SUA did not vary significantly according to age (P for interaction = 0.172) nor gender (P for interaction = 0.597).

In addition, the ROC curve showed that the uric acid levels were a fair predictor of functional disability (AUC = 0.67; 95% CI = 0.58-0.77; *P* = .025); the best cutoff level to predict disability was above 5.5 mg/dL.

Adopting this cutoff, 100 (58%) participants were confirmed to be functionally disabled.

## Discussion

Results our study indicate that SUA levels are associated with disability in the instrumental activities of daily living in community-dwelling elders; SUA levels above 5.5 mg/dL best predict disability. This finding adds information on the complex association of SUA levels with cerebral and muscular integrity and functioning, which in turn underlie frailty and disability. Indeed, whether predominantly relying on complex cognitive tasks, such as using the phone or managing finances, or physical performance, such as housekeeping or shopping for groceries, completing the instrumental activities of daily living always needs adequate levels of cognitive function and physical ability [6].

Although higher SUA levels have been associated with increased muscle strength and better cognitive functioning [7], such favorable effects have generally been observed among subjects whose mean age was below 70 years [8]. Indeed, the direction of the association of SUA with cognitive and muscle functioning, as well as with the development of frailty, seem to be quite different in older populations, like that enrolled in the present study.

According to available evidence, uric acid is a double-edged sword, as increased serum and/or tissue levels of this compound have been associated with endothelial dysfunction and vasoconstriction, and mitochondrial dysfunction [9]; reduced nitric oxide tissue levels have also been associated with renal and myocardial fibrosis [10]. In addition, increased SUA levels are associated with activation of chronic low-grade inflammation.

Also, increased serum uric acid levels are associated with activation of chronic low-grade inflammation, chiefly through the NLRP3 inflammasome [11]. This is a multiprotein complex that, once activated by metabolites like uric acid, reactive oxygen species or cholesterol, promotes pro-caspase 1 maturation and then proceeds to cleave pro-IL-1β and pro-IL-18 into mature IL-1β and IL-18. Interestingly, activation of the NLRP3 inflammasome is considered central to the onset and progression of “inflammaging”, a condition characterized by self-perpetuating low-grade inflammation that drives a series of alterations (including atherosclerosis, myocardial fibrosis, sarcopenia, and neuronal dysfunction with consequent apoptosis) which are a hallmark of the transition from normal aging to frailty and disability.

Results of a large Italian cross-sectional study suggested a positive association between high circulating levels of uric acid and prevalent dementia [12]. In the Rotterdam Scan Study, hyperuricemic patients exhibited increased grades of white matter atrophy as compared with normouremic subjects [13].

A gender-specific effect has been hypothesized for this association. However, our results did not confirm such hypothesis, possibly because several biological differences between gender tend to fade in the most advanced age strata of populations [14].

Eventually, analyses of the NAHNES III database indicated an association of SUA with sarcopenia [15], which in turn is a keystone of frailty and disability.

A strength of our study is the inclusion of a whole, unselected population of community-dwelling elderly, with comprehensive information regarding lifestyle habits, comorbid conditions, medications, and objective clinical and laboratory parameters. However, due to its cross-sectional design, this study does not allow to establish any cause-effect relationships. In particular, we were unable to ascertain whether reductions of SUA levels might affect the onset or progression of disability.

Overall, our results warrant further studies designed to assess the effect of pharmacological lowering of serum and tissue uric acid levels on the development and progression of disability in elderly populations, aiming at achieving serum levels below 6 mg/dL. Also, SUA levels above 5.5 mg/dL might be used in clinical practice to identify older subjects in whom physical exercise and appropriate diet should be implemented to help preventing the development of disability.
